# Supplementary material for: Simultaneously Enhancing the Efficiency and Stability of Perovskite Solar Cells by Using P3HT/PEDOT:PSS as a Double Hole Transport Layer
Source: Nanomaterials (Basel). 2024 Sep 11;14(18):1476. doi: 10.3390/nano14181476 (PMC11434247; doi:10.3390/nano14181476)
Supplement: Supplementary file 1 [file nanomaterials-14-01476-s001.zip › nanomaterials-3155037-supplementary.pdf]

Supporting information

Simultaneously enhancing efficiency and stability of perovskite solar cells by using P3HT/PEDOT:PSS as double hole transport layer

Xiude Yang [1,†], Minghao Luo [1,†], Qianqian Zhang [1], Haishen Huang [1], Yanqing Yao [1], Yuanlin Yang [2], Ying Li [2], Wan Cheng [2] and Ping Li [1,*]

[1] School of Physics and Electronic Science, Zunyi Normal College, Zunyi 563006, China

[2] College of Physics and Electronic Engineering, Chongqing Normal University, Chongqing 401331, China

* Correspondence: lip19870212@126.com

† These authors contributed equally to this work.

Table S1 Summarizes and compares the PCE of hydrophobic polymers in the conventional HTLs of perovskite solar cells.

Device	hydrophobic polymers	PCE	Years
ITO/poly TPD/Perovskite/$PC_{61}BM$/C_{60}/BCP/Au	Poly TPD	15.15 %[1]	2019
ITO/PEDOT:PSS/Perovskite/$PC_{60}BM$/Ag	PTAA	16.94 %[2]	2019
ITO/PEDOT:PSS/PTAA/Perovskite/$PC_{60}BM$/Ag		19.04 %[2]	
ITO/PTAA/Perovskite/$PC_{60}BM$/BCP/Ag	PTAA	6.9 %[3]	2021
ITO/PEDOT:PSS/Perovskite/$PC_{60}BM$/BCP/Ag		10.1 %[3]	
ITO/PTAA/Perovskite/C_{60}/BCP/Ag	PTAA:PASQ-IDT	19.74 %[4]	2022
ITO/PTAA:PASQ-IDT/Perovskite/C_{60}/BCP/Ag		21.33 %[4]	
ITO/PTAA/Perovskite/PCBM/BCP/Ag	PEO-TPAB	18.33 %[5]	2023
ITO/PTAA/PEO/Perovskite/PCBM/BCP/Ag		20.11%[5]	
ITO/PTAA/PEO-TPAB/Perovskite/PCBM/BCP/Ag		21.62 % [5]	
ITO/PTAA(PhMe)/Perovskite/C_{60}/BCP/Ag	PTAA(PhMe) and PTAA(Py)	19.35 %[6]	2023
ITO/PTAA(Py)/Perovskite/C_{60}/BCP/Ag		20.53 %[6]	
ITO/PEDOT:PSS/Perovskite/PCBM/BCP/Ag	P3HT	17.04 %	This work
ITO/PEDOT:PSS/P3HT/Perovskite/PCBM/BCP/Ag		19.78 %	

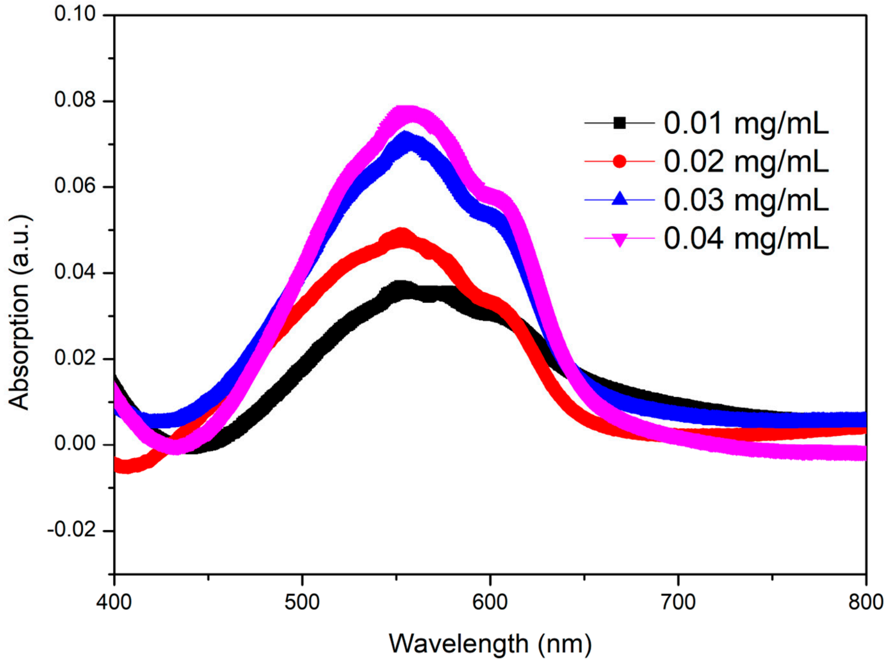

Fig.S1. The absorption of various concentration of P3HT.

1. J Höcker, D Kiermasch, P Rieder, K Tvingstedt, A Baumann, V Dyakonov. Efficient Solution Processed $CH_3NH_3PbI_3$ Perovskite Solar Cells with PolyTPD Hole Transport Layer[J]. Z. Naturforschung A. 74(2019) 665-672. https://doi.org/10.1515/zna-2019-0127.
2. M Wang, H Wang, W Li, X Hu, K Sun, Z Zang. Defect Passivation Using Ultrathin PTAA Layers for Efficient and Stable Perovskite Solar Cells with a High Fill Factor and Eliminated Hysteresis[J]. J. Mater. Chem. A. 7(2019) 26421-26428. https://doi.org/10.1039/C9TA08314F.
3. H Mehdi, M Matheron, A Mhamdi, S Cros, A Bouazizi. Effect of the Hole Transporting Layers on the Inverted Perovskite Solar Cells[J]. J Mater Sci Mater Electron. 32(2021) 21579-21589. https://doi.org/10.1007/s10854-021-06666-z.
4. F Wu, Q Xiao, X Sun, T Wu, Y Hua, L Zhu. Hole Transporting Layer Engineering Via a Zwitterionic Polysquaraine Toward Efficient Inverted Perovskite Solar Cells[J]. Chem. Eng. J. 445 (2022): 136760. https://doi.org/10.1016/j.cej.2022.136760.
5. J Dai, J Xiong, N Liu, Z He, Y Zhang, S Zhan, B Fan, W Liu, X Huang, X Hu, D Wang, Y Huang, Z Zhang, J Zhang. Synergistic Dual-interface Modification Strategy for Highly Reproducible and Efficient PTAA-based Inverted Perovskite Solar Cells[J]. Chem. Eng. J. 453 (2023): 139988. https://doi.org/10.1016/j.cej.2022.139988.
6. Y Wang, J Song, L Chu, Y Zang, Y Tu, J Ye, Y Jin, G Li, Z Li, W Yan. Buried Solvent Assisted Perovskite Crystallization for Efficient and Stable Inverted Solar Cells[J]. J. Power Sources. 558 (2023): 232626. https://doi.org/10.1016/j.jpowsour.2023.232626.
